# The development of orthodontic elastomeric ligature with sustained fluoride-releasing for the prevention of white spot lesions: an *in vitro* feasibility study

**DOI:** 10.3389/fbioe.2025.1671922

**Published:** 2026-01-20

**Authors:** Kativa Strickland, Peter Buschang, Ying Liu, Amal Noureldin, Reginald Taylor, Matthew Kesterke, Chi Ma, Yan Jing

**Affiliations:** 1 Department of Orthodontics, Texas A&M College of Dentistry, Dallas, TX, United States; 2 Department of Dental Public Health Sciences, Texas A&M College of Dentistry, Dallas, TX, United States; 3 Scottish Rite for Children, Dallas, TX, United States; 4 Department of Orthopaedic Surgery, University of Texas Southwestern Medical Center, Dallas, TX, United States

**Keywords:** fluoride, white spot lesions (WSLs), orthodontics, orthodontic elastomeric ligatures (O-rings), polycaprolactone (PCL)

## Abstract

**Objectives:**

Demineralization of enamel is a major challenge during and after fixed orthodontic treatment. Fluoride can strengthen the enamel and reduce the occurrence of white spot lesions (WSLs). Current fluoride-releasing products exhibit a short-term release due to initial burst effect, which severely limits clinical effectiveness. O-rings are orthodontic elastomeric ligatures used to support the attachment of arch-wire to each bracket. This study aimed to develop a simple method to coat the O-rings for long-lasting fluoride release.

**Methods:**

Calcium fluoride (CaF_2_) was coated on the commercial O-rings via a dip and dry method using a coating medium composed of a solution of polycaprolactone (PCL) with CaF_2_ microcrystals. To optimize the fluoride release, the coating media with different concentrations of PCL (2.5%, 5%, and 10%) solution were applied, and the fluoride release was measured for 7 weeks. The morphology and elemental abundance of the coatings were characterized by energy-dispersive X-ray spectroscopy. The resilience of the modified O-rings was evaluated by a standard tensile program.

**Results:**

A thicker coating with a higher elemental abundance of fluoride was achieved by increasing the PCL concentration in the coating medium. The average fluoride release rates of the 2.5%, 5%, and 10% groups in the seventh week were 0.69 μg F^−^/ring/day, 6.54 μg F^−^/ring/day, and 6.97 μg F^−^/ring/day, respectively. 5% and 10% groups showed long-term and linear release within the therapeutic range, while the 2.5% group fell below the range from the sixth week.

**Conclusion:**

Our study demonstrated Ca-F O-rings displayed sustained fluoride release under *in vitro* conditions, indicating potential clinical relevance for reducing WSLs during orthodontic treatment. This work represents an early-stage feasibility study and warrants further validation with larger-scale and *in vivo* conditions.

## Introduction

White spot lesions (WSLs) are opaque, chalky white areas on tooth enamel caused by demineralization (Lopes et al., 2024). WSLs pose substantial esthetic problems for patients (Ogaard, 1989). Approximately 50% of orthodontic patients develop WSLs on at least one tooth (Mizrahi, 1982). In some severe cases, the occurrence of WSLs during orthodontic treatment may necessitate premature debonding to prevent further damage to the enamel (Baturina et al., 2010). Such problems have caused clinicians to search for a solution to prevent orthodontic-associated demineralization.

Tooth enamel is primarily composed of hydroxyapatite, which is susceptible to acid dissolution due to pH fluctuations during orthodontic treatment (Shankarappa et al., 2024). When fluoride ions are present, they can partially replace hydroxyl groups in hydroxyapatite, leading to the formation of fluorapatite, a mineral phase with extremely low solubility. This transformation effectively reduces the critical pH for enamel demineralization from approximately 5.5 to 4.5 (Buzalaf et al., 2011). Moreover, fluoride ions facilitate remineralization by stabilizing calcium and phosphate ions near the tooth surface, thereby enhancing the redeposition of minerals within demineralized enamel lesions (Cochrane et al., 2010; Gonzalez-Cabezas et al., 2018). It has been reported that 0.05–1 ppm fluoride in daily exposure can effectively prevent enamel demineralization (Baturina et al., 2010; Margolis et al., 1986).

Fluoride-containing products were developed to enhance fluoride uptake by tooth structures and prolong the sustained release of fluoride. However, most fluoride delivery vehicles, such as fluoride varnishes, sealants, orthodontic adhesives, and glass ionomer cements, exhibit an initial burst release of fluoride within the first few days of application, followed by a rapid decline in release rate (Perrini et al., 2016; Nascimento et al., 2016; Piesiak-Panczyszyn et al., 2023). This results in insufficient fluoride concentrations in saliva over time, which significantly limits the duration of effectiveness of these materials in preventing WSLs.

The burst effect is attributed to the high solubility of fluoride sources such as stannous fluoride or sodium fluoride (Piesiak-Panczyszyn et al., 2023; Wiltshire, 1996; O'Dwyer et al., 2005). In contrast, calcium fluoride (CaF_2_) exhibits low solubility, allowing only a small amount to dissolve at a time. The equilibrium between the solid and dissolved ions can maintain a steady release rate when fluoride is consumed. CaF_2_ has been recognized as a fluoride reservoir that aids in the repair of demineralized enamel (ten Cate, 1997; Yi et al., 2019). However, the *in situ* formation of CaF_2_ during dental care is limited due to the low availability of calcium or fluoride ions in saliva (Vogel, 2011). This limitation can be overcome by incorporating exogenous calcium fluoride particles into dental care products.

O-rings are small elastomeric ligatures used to secure the archwire to orthodontic brackets and are routinely replaced at each adjustment visit, typically every 4–6 weeks. It has long been considered a potential compliance-free carrier for therapeutic agents. Early fluoridated O-rings introduced in the 1990s and early 2000s demonstrated high initial fluoride release but were unable to sustain therapeutic levels beyond 1 week, leading to their discontinuation (Wiltshire, 1996; O'Dwyer et al., 2005). More recently, advances in coating technologies have revived interest in O-rings as delivery platforms. Coatings incorporating silver nanoparticles, bacterial nanocellulose, or zinc oxide nanoparticles have shown long-lasting antibacterial activity, reduced plaque accumulation, and decreased caries development (Eskandari et al., 2024; Hernandez-Gomora et al., 2017; Schubert et al., 2024; Hemashree and Padmanabhan, 2025). These studies demonstrate that O-rings can successfully support controlled, sustained release of bioactive materials when appropriate coating strategies are used.

Polycaprolactone (PCL) is approved for use in surgical implants and drug delivery devices for tissue engineering and regenerative medicine applications due to its biosafety. PCL is partially crystalline and has a low glass transition temperature of −59 to −64 °C. This grants the PCL and its composites extraordinary tensile extensibility (Chen et al., 2010), allowing it to serve as a potential carrier for microparticles and stabilize the coating layer on the surface of O-ring during stretching (Pawar et al., 2023).

In this study, which represents the first step in a series of projects, we established a simple dip and dry method to coat CaF_2_ microcrystals onto commercial O-rings. The goal was to develop a fluoride-releasing O-ring capable of sustaining therapeutically relevant fluoride over a clinically meaningful interval between orthodontic follow-up visits (up to 7 weeks). Additionally, we verified the chemical composition of the coating and evaluated the mechanical performance of the modified O-rings.

## Materials and methods

### Preparation of fluoridated O-rings

**FIGURE 1:**
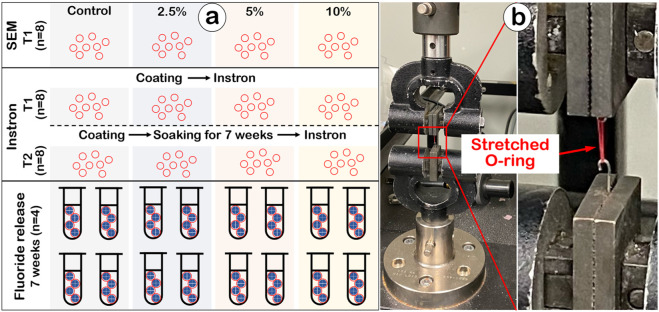
**(a)** The sample allocations for SEM, mechanical performance (Instron) and fluoride release. **(b)** The mechanical performance of the Ca-F O-rings was evaluated by Instron. T1, after O-ring preparation; T2, after 7-week soaking.

The Ca-F O-rings were prepared by a simple dip and dry coating method. First, the coating medium was prepared by mixing solutions A and B with 1 to 1 (v/v). Specifically, solution A was prepared by dispersing CaF_2_ micro crystals in acetone (400 mg/mL). Three different concentrations of solution B were prepared by dissolving 5%, 10%, or 20% of PCL (Sigma, 440744) in acetone. 0.5 mL of solution A and B were mixed to obtain three Ca-F coating media: 2.5%, 5% or 10% PCL with CaF_2_. Second, the ordinary O-rings (American Orthodontics, WI) were incubated into a coating medium for one second and transferred into a dry oven for slow solvent evaporation for 1 h. After that, all the coated O-rings were moved into a vacuum chamber for additional drying for 24 h. A total of four groups of O-rings were set: 1) ordinary O-rings (control group); 2) Ca-F O-rings coated with 2.5% PCL (2.5% group); 3) Ca-F O-rings coated with 5% PCL (5% group); and 4) Ca-F O-rings coated with 10% PCL (10% group). For the fluoride-release experiment, four O-rings were pooled into a single tube to reduce variability among individual O-rings, and the mean fluoride concentration from each tube was treated as one experimental unit for analysis. A total of four tubes (n = 4) were analyzed per group. For Instron mechanical testing and SEM analyses, eight O-rings were used per group for each test (Figure 1a). Because mechanical testing could cause irreversible elongation and material fatigue, independent sets of O-rings were used at each time point. These sample sizes were chosen to ensure sufficient replication for a feasibility-level materials study while remaining consistent with standard practice in exploratory biomaterials research.

### Scanning electron microscopy

Scanning electron microscopy (SEM) was used to characterize the morphology of the O-rings and measure the cross-sectional thickness of the PCL layer after O-ring preparation (T1). The Ca-F O-rings were rinsed in liquid nitrogen for 3 min and then snapped into pieces to expose the cross-sectional surface. The surface was coated with gold for SEM scanning (Jing et al., 2018; Jing et al., 2017). To determine the amount of fluoride and calcium ions in the PCL layers, energy-dispersive x-ray spectroscopy (EDX) associated with SEM (EDX/SEM) mapping was used to detect the abundance of fluoride and calcium ions, and to quantify the mass ratio of calcium and fluoride in each group as previously described (Jing et al., 2017). This provided the means to determine if calcium and fluoride ions were successfully incorporated into the PCL layer on the O-ring surface. The cross-section of each O-ring was divided into four-quarters, and three points were randomly selected from each quarter. The average of a total of 12 points was used to represent the thickness of the PCL layer and the EDX results for each O-ring.

### *In vitro* test of fluoride release

To test fluoride release, all O-rings were placed on brackets for the maxillary lateral incisor (American Orthodontics, WI) to eliminate the influence of stretching force on fluoride release (O'Dwyer et al., 2005). Every four O-rings were immersed in 10 mL of incubation medium (distilled water) in a 50 mL centrifuge tube. Each group consisted of four tubes, containing a total of 16 O-rings per group. All tubes were incubated at 37 °C throughout the release period. Fluoride concentration was measured using an Ion Selective Electrode (ISE) meter (Thermo Scientific Orion Dual Star). Measurements were taken at ten time points: Day 1, Day 3, Day 5, Week 1, Week 2, Week 3, Week 4, Week 5, Week 6, and Week 7. The ISE meter was calibrated at each measurement point following the manufacturer’s instructions. After each measurement, the O-rings were transferred to new tubes containing 10 mL of fresh incubation medium until the final time point.

### Mechanical test

The mechanical performance of the Ca-F O-rings was evaluated using an Instron testing machine (Instron® 5567, Figure 1b). Tests were conducted at two time points: before soaking in distilled water (T1) and after soaking for 7 weeks (T2). Testing parameters followed ISO 21606:2007 guidelines (IOS, 2007). Briefly, each specimen was stretched at a rate of 100 mm/min to four times its outer diameter (OD) and held for 5 s. The O-ring was then returned to three times its OD at the same rate and held for 30 s, during which the final tensile force was recorded. If the O-ring could not be stretched to four times its OD, tensile failure was noted. The maximum force (N) at the time of breakage was recorded; if no breakage occurred, the final tensile force (N) at the end of the test was documented.

### Statistical analysis

All statistical analyses were performed using GraphPad Prism. Data normality and homogeneity of variances were confirmed using the Shapiro-Wilk and Brown-Forsythe tests. One-way ANOVA was used to analyze SEM, EDX, and mechanical testing data, followed by Tukey’s *post hoc* test. Two-way ANOVA was used to analyze fluoride release rates over the 7-week period, followed by Holm’s *post hoc* test. An unpaired t-test was applied to compare mechanical properties between T1 and T2 because different specimens were tested at each time point. Each fluoride measurement was based on three technical replicates using the ISE meter, with an intraclass correlation coefficient (ICC) of 99.9%.

## Results

### Surface morphological structure of Ca-F O-ring

**FIGURE 2:**
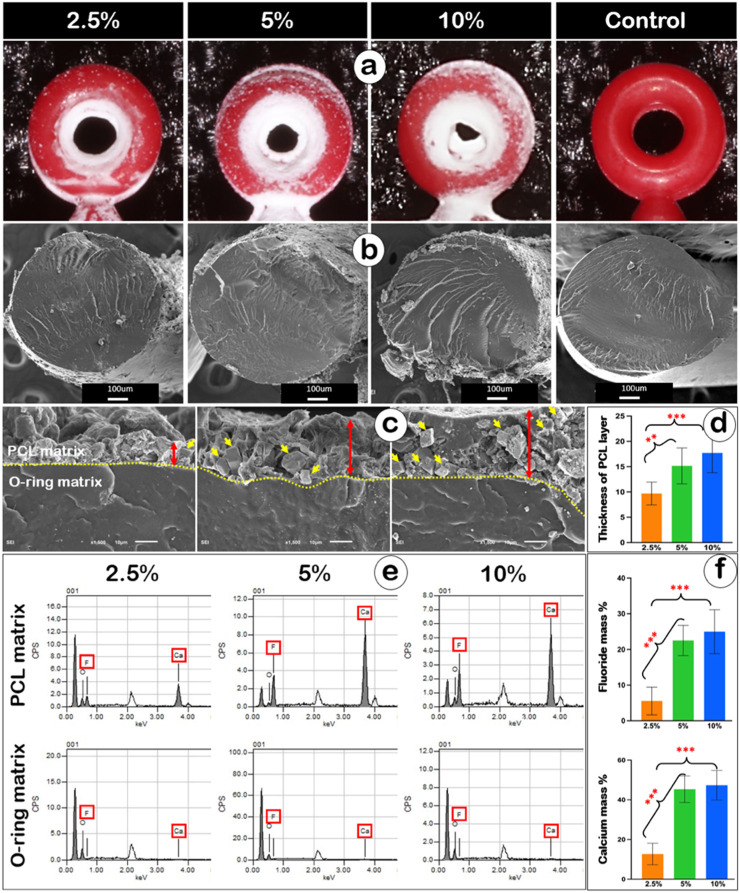
PCL carrying CaF_2_ microcrystals successfully coated onto the O-ring. **(a)** Optical images showing Ca-F O-rings in the 2.5%, 5%, and 10% groups, along with the non-coated O-ring. **(b)** Low-magnification SEM images showing cross sections of the 2.5%, 5%, and 10% groups, and the non-coated O-ring. **(c)** High-magnification SEM images showing the PCL coating layer on the O-ring matrix (red double arrows indicate the PCL layers) in the 2.5%, 5%, and 10% groups. **(d)** Bar graph showing the quantitative analysis of coating thickness in Ca-F O-rings. One-way ANOVA, **p < 0.01; ***p < 0.001. **(e)** EDX spectra showing absorbance peaks of fluoride and calcium ions detected in the PCL coating layer but not in the O-ring bulk matrix. **(f)** Bar graphs showing the quantitative results of fluoride and calcium element mass percentages in the three groups of Ca-F O-rings. One-way ANOVA, ***p < 0.001.

The three sets of Ca-F O-rings exhibited a distinct white PCL coating layer on their surfaces (Figure 2a). At lower magnification (T1), SEM images showed a PCL layer deposited on the matrix of Ca-F O-rings, whereas the surface of regular O-rings appeared smooth and retained their original morphology (Figure 2b). At higher magnification (Figure 2c), SEM images revealed a gradual increase in PCL layer thickness as the concentration increased from 2.5% (9.70 ± 2.25 μm) to 5% (15.15 ± 3.55 μm) and 10% (17.73 ± 3.91 μm), consistent with the quantitative measurements (Figure 2d). With increasing PCL content, more CaF_2_ crystals were embedded within the PCL matrix (Figure 2c, yellow arrows).

EDX analysis revealed absorbance peaks for fluoride and calcium ions within the PCL matrix of the 2.5%, 5%, and 10% groups, whereas no fluoride or calcium ions were detected in the control O-ring matrix (Figure 2e). Quantitative analysis showed that the elemental mass percentage of fluoride increased significantly with higher PCL concentrations (2.5% group: 5.55% ± 3.89%; 5% group: 22.49% ± 4.23%; 10% group: 24.97% ± 6.15%; Figure 2f, upper panel). Calcium followed a similar trend (2.5% group: 12.70% ± 5.40%; 5% group: 45.36% ± 6.70%; 10% group: 47.35% ± 7.45%; Figure 2f, lower panel).

These data demonstrate the successful integration of calcium fluoride into the PCL layer on the O-rings. Both the thickness of the PCL layer and the fluoride ion content increased with higher PCL concentrations.

### *In vitro* fluoride release of Ca-F O-rings

**FIGURE 3:**
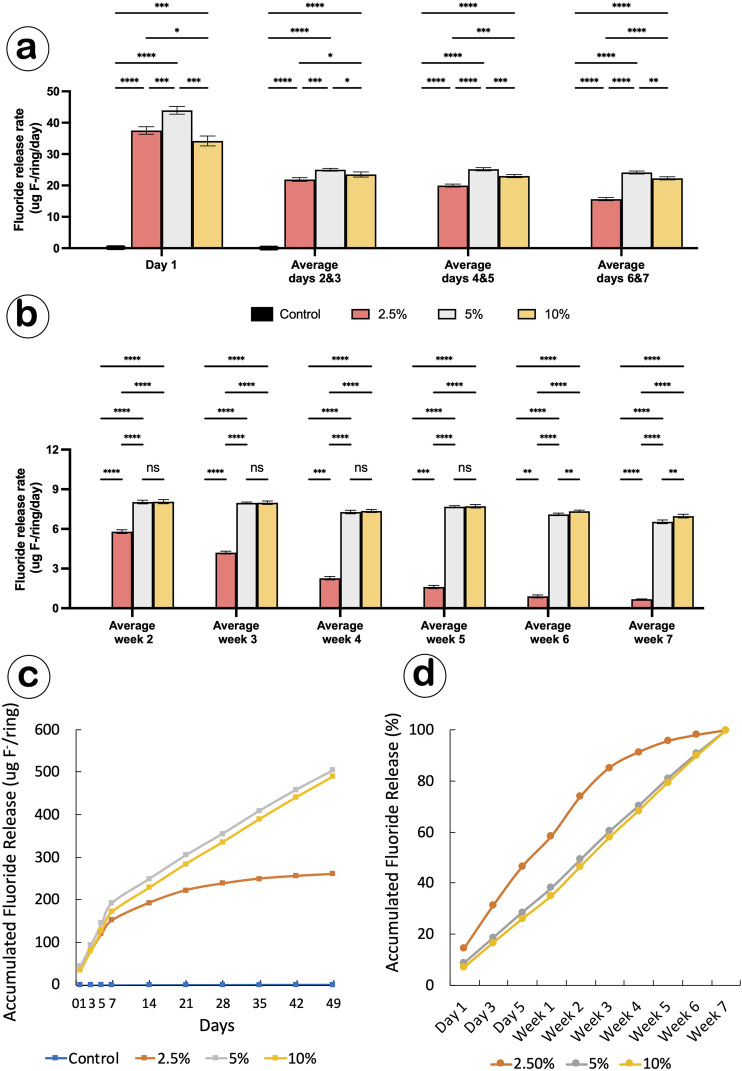
Ca-F O-rings produced a sustained release of fluoride. **(a,b)** Bar graphs showing the average fluoride release rate of Ca-F O-rings during the first week **(a)** and for the subsequent weeks **(b)**; Two-way ANOVA, *p < 0.05; **p < 0.01; ***p < 0.001; ****p < 0.0001. **(c,d)** Cumulative fluoride release from Ca-F O-rings presented as total amount **(c)** and percentage **(d)** over 7 weeks.

During the first week, we examined the fluoride burst effect by analyzing the release rates on Day 1 and averaging the rates for Days 2–3, 4–5, and 6–7. In all experimental groups, the release rate significantly decreased from Day 1 to Days 2–3. Fluoride release in the control group was minimal, and significant differences were observed between groups during the first week (Table 1; Figure 3a).

**TABLE 1:** Daily fluoride release rate in the first week (μg F^−^/ring/day).

Groups	Mean 1	Mean 2	P value
Day 1			
Control vs. 2.5%	0.1699	37.52	<0.0001
Control vs. 5%	0.1699	43.96	<0.0001
Control vs. 10%	0.1699	34.15	0.0001
2.5% vs. 5%	37.52	43.96	0.0006
2.5% vs. 10%	37.52	34.15	0.0162
5% vs. 10%	43.96	34.15	0.0003
Average days 2 and 3			
Control vs. 2.5%	0.0354	21.9	<0.0001
Control vs. 5%	0.0354	25	<0.0001
Control vs. 10%	0.0354	23.46	<0.0001
2.5% vs. 5%	21.9	25	0.0006
2.5% vs. 10%	21.9	23.46	0.0467
5% vs. 10%	25	23.46	0.0467
Average days 4 and 5			
Control vs. 2.5%	0.0193	19.97	<0.0001
Control vs. 5%	0.0193	25.18	<0.0001
Control vs. 10%	0.0193	22.97	<0.0001
2.5% vs. 5%	19.97	25.18	<0.0001
2.5% vs. 10%	19.97	22.97	0.0003
5% vs. 10%	25.18	22.97	0.0009
Average days 6 and 7			
Control vs. 2.5%	0.0246	15.65	<0.0001
Control vs. 5%	0.0246	24.14	<0.0001
Control vs. 10%	0.0246	22.24	<0.0001
2.5% vs. 5%	15.65	24.14	<0.0001
2.5% vs. 10%	15.65	22.24	<0.0001
5% vs. 10%	24.14	22.24	0.0016

We then assessed the mid- and long-term release profile. Daily release rates from weeks 2 to 7 were calculated by averaging the weekly release. The release remained consistent at subsequent time points in the 5% and 10% groups but not in the 2.5% group (Table 2; Figure 3b). By the seventh week, the average fluoride release rates were 0.69, 6.54, and 6.97 μg F^−^/ring/day for the 2.5%, 5%, and 10% groups, respectively.

**TABLE 2:** Average daily fluoride release rate by week (μg F^−^/ring/day).

Groups	Mean 1	Mean 2	P value
Average week 2			
Control vs. 2.5%	0.0066	5.795	<0.0001
Control vs. 5%	0.0066	8.042	<0.0001
Control vs. 10%	0.0066	8.063	<0.0001
2.5% vs. 5%	5.795	8.042	<0.0001
2.5% vs. 10%	5.795	8.063	<0.0001
5% vs. 10%	8.042	8.063	0.8489
Average week 3			
Control vs. 2.5%	0.0053	4.211	<0.0001
Control vs. 5%	0.0053	7.976	<0.0001
Control vs. 10%	0.0053	7.982	<0.0001
2.5% vs. 5%	4.211	7.976	<0.0001
2.5% vs. 10%	4.211	7.982	<0.0001
5% vs. 10%	7.976	7.982	0.9388
Average week 4			
Control vs. 2.5%	0.0048	2.266	0.0001
Control vs. 5%	0.0048	7.28	<0.0001
Control vs. 10%	0.0048	7.354	<0.0001
2.5% vs. 5%	2.266	7.28	<0.0001
2.5% vs. 10%	2.266	7.354	<0.0001
5% vs. 10%	7.28	7.354	0.4344
Average week 5			
Control vs. 2.5%	0.0045	1.607	0.0003
Control vs. 5%	0.0045	7.682	<0.0001
Control vs. 10%	0.0045	7.714	<0.0001
2.5% vs. 5%	1.607	7.682	<0.0001
2.5% vs. 10%	1.607	7.714	<0.0001
5% vs. 10%	7.682	7.714	0.6792
Average week 6			
Control vs. 2.5%	0.0053	0.8949	0.001
Control vs. 5%	0.0053	7.098	<0.0001
Control vs. 10%	0.0053	7.342	<0.0001
2.5% vs. 5%	0.8949	7.098	<0.0001
2.5% vs. 10%	0.8949	7.342	<0.0001
5% vs. 10%	7.098	7.342	0.0092
Average week 7			
Control vs. 2.5%	0.0047	0.6857	<0.0001
Control vs. 5%	0.0047	6.539	<0.0001
Control vs. 10%	0.0047	6.97	<0.0001
2.5% vs. 5%	0.6857	6.539	<0.0001
2.5% vs. 10%	0.6857	6.97	<0.0001
5% vs. 10%	6.539	6.97	0.0051

The cumulative release curves illustrate fluoride release over the 7-week testing period. A linear release was observed in the 5% and 10% groups, whereas the release rate plateaued after 3 weeks in the 2.5% group (Figure 3c). The total amounts of fluoride released per ring were 260.8, 504.9, and 489.5 μg for the 2.5%, 5%, and 10% groups, respectively. The cumulative release percentage curves showed that approximately 60% of fluoride was released by the first week and over 90% by week 4 in the 2.5% group. In contrast, the 5% and 10% groups released only 38% and 35% of fluoride by the first week, respectively, with the release percentage increasing steadily until the end of the experiment (Figure 3d).

Taken together, the Ca-F coating provides sustained fluoride release with a low-level burst. The 5% and 10% Ca-F O-ring groups exhibited long-lasting fluoride release over the 7-week experimental period.

### Mechanical performance of Ca-F O-rings

**FIGURE 4:**
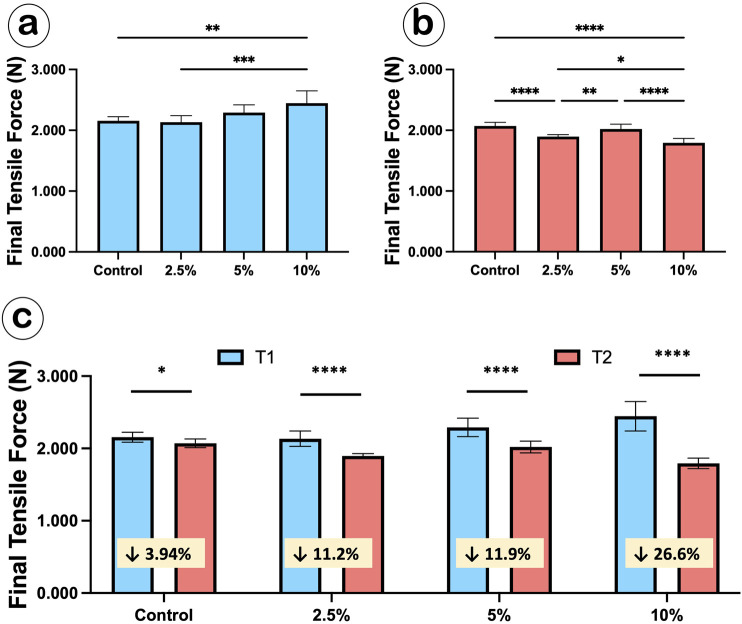
The elastic performance of Ca-F O-rings before and after *in vitro* release medium incubation. **(a,b)** Bar graphs showing the final tensile forces of Ca-F O-rings before (T1, **(a)**) and after 7-week release medium incubation (T2, **(b)**). One-way ANOVA, *p < 0.05; **p < 0.01; ***p < 0.001, ****p < 0.0001; **(c)** Bar graph showing the comparison of elastic performance between T1 and T2 in the four groups of O-rings. t-test, *p < 0.05; ****p < 0.0001.

Tensile failure was not observed in any group at any time point. At T1, the final tensile force in the 2.5% group (2.13 ± 0.11 N) slightly decreased compared to controls (2.16 ± 0.07 N), while the 5% (2.29 ± 0.13 N) and 10% (2.45 ± 0.20 N) groups showed a moderate and significant increase compared to controls, respectively (Figure 4a). By T2, the final tensile forces of 2.5% (1.90 ± 0.03 N), 5% (2.02 ± 0.08 N) and 10% (1.79 ± 0.07 N) groups all decreased compared to controls (2.07 ± 0.06 N), with statistically significant reductions noted in the 2.5% and 10% groups (Figure 4b).

Comparing T1 to T2, both control and Ca-F O-rings displayed statistically significant decreases of final tensile forces (Figure 4c). Within the groups, percentage decreases of final tensile forces were −3.94% for controls, −11.2% for the 2.5% group, −11.9% for the 5% group, and −26.6% for the 10% group.

**FIGURE 5:**
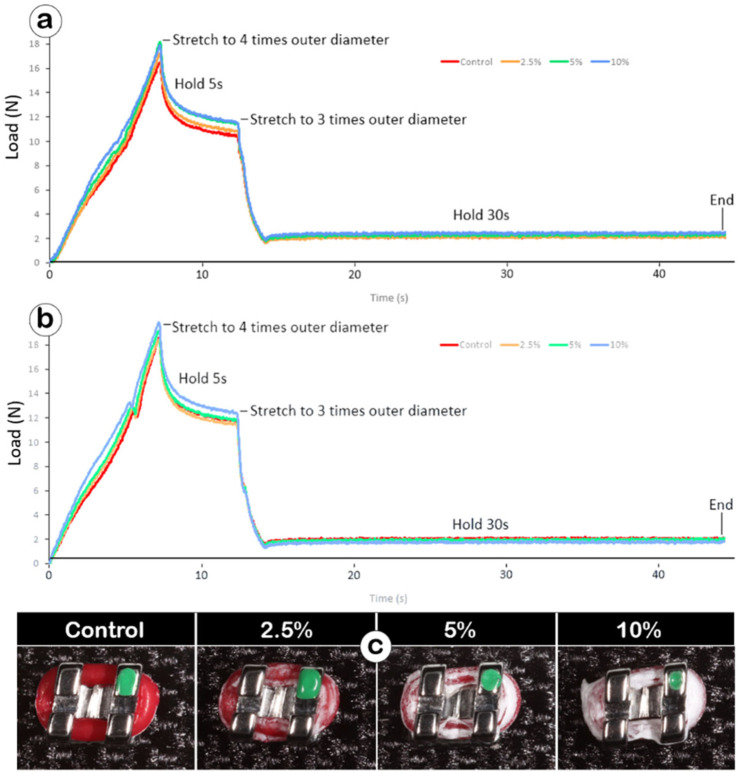
**(a,b)** The representative elastic curves after coating **(a)** and after 7-week release **(b)**; **(c)** the appearance of the control and Ca-F O-rings after 7-week soaking.

Throughout the mechanical test, all groups exhibited similar elastic curves at both T1 (Figure 5a) and T2 (Figure 5b). Additionally, all Ca-F O-rings maintained their structural integrity on the brackets at T2 (Figure 5c). Taken together, the Ca-F O-rings maintained acceptable orthodontic performance requirements.

## Discussion

In this study, we developed a straightforward dip and dry method to coat commercial O-rings with CaF_2_ microcrystals, optimizing for prolonged fluoride release without significantly disrupting their mechanical performance. This approach holds promising potential application in preventing WSLs during orthodontic treatment.

The Ca-F O-rings demonstrated sustained fluoride release in distilled water. The 5% group released 6.54–43.6 μg F^−^/ring/day, and the 10% group released 6.97–34.15 μg F^−^/ring/day. These values exceed the reported therapeutic range (1.2–2.8 μg F^−^/ring/day) yet remain well below the toxic threshold (51 μg F^−^/ring/day), a level associated with possible enamel fluorosis (Baturina et al., 2010). Moreover, the 5% and 10% groups maintained nearly linear fluoride release and remained above the therapeutic level from the second through the seventh week. Collectively, the 5% and 10% Ca-F O-rings demonstrated long-term, sustained fluoride release compatible with the typical 4 to 6-week orthodontic follow-up intervals for fixed appliance adjustments (Tsichlaki et al., 2016).

Compared with previous studies, our method achieved significant improvements in sustaining fluoride release and minimizing the burst effect. Prior reports using Fluor-I-Tie O-rings showed a sharp decline from 9.68 to 0.79 μg F^−^/ring/day within 7 days, with average levels below the therapeutic range (O'Dwyer et al., 2005). Other formulations released 35%–63% of total fluoride within the first week (Wiltshire, 1996) or produced excessive burst release exceeding the toxic limit (115 μg F^−^/ring on day 1) before rapidly dropping below therapeutic levels (Baturina et al., 2010). In contrast, the 5% and 10% groups in the present study released less than 10% of total fluoride on day one and under 40% by week one, maintaining therapeutic fluoride release throughout the 7-week period. This sustained release with a controlled burst profile may better prevent enamel demineralization and reduce the incidence of WSLs during orthodontic treatment.

Two key factors contributed to the improvement in sustained fluoride release and the reduction of the burst effect. The PCL coating was one of these factors and played dual roles. First, PCL served as a carrier for the CaF_2_ microcrystals. Higher PCL concentrations produced a more viscous coating medium and consequently a thicker coating layer with more CaF_2_ loading. SEM and EDX analyses confirmed that both the PCL layer thickness and the quantities of incorporated fluoride and calcium increased progressively from the 2.5% to the 5% and 10% groups. Due to the low CaF_2_ loading, the 2.5% group showed a markedly lower fluoride release rate compared to the 5% and 10% groups, while the latter maintained a high and consistent fluoride release over the 7-week period. Second, due to the hydrophobic nature of PCL, thicker coatings reduced direct exposure of CaF_2_ to the aqueous environment. As a result, the 10% group exhibited lower fluoride release rates than the 5% group during the early and middle release periods.

The other key factor contributing to the sustained fluoride release design is the selection of CaF_2_ microcrystals as the fluoride reservoir. CaF_2_ has low solubility, allowing only a small amount to dissolve at a time. This property maintains fluoride concentrations near saturation, preventing burst release and enabling gradual, sustained fluoride delivery. In contrast, highly soluble fluoride compounds, such as sodium fluoride and stannous fluoride, produce a pronounced initial burst effect (Mattick et al., 2001; Banks et al., 2000). Moreover, CaF_2_ can modulate fluoride release in response to environmental pH according to Le Chatelier’s principle. Under acidic conditions, the dissolution equilibrium shifts to release more fluoride ions, thereby protecting enamel from demineralization (Kumari et al., 2019). These features make calcium fluoride a promising candidate as a fluoride-reservoir material for the prevention of WSLs. The present study highlights the potential of O-rings as carriers for the slow and consistent delivery of calcium fluoride, compensating for the limited availability of calcium ions in saliva (Vogel, 2011).

The modified O-rings maintained their elastic strength, and no tensile failures were observed in any Ca-F O-rings after coating (T1) or 7 weeks of soaking (T2). This preservation of elasticity may be attributed to the similar elastic modulus between the Ca-F-PCL matrix and the original O-ring material. The 10% group exhibited a significant increase in final tensile strength upon stretching, likely due to the increased overall diameter resulting from thicker PCL coating. The experimental groups showed an 11%–26% decrease in final tensile force from T1 to T2, which falls within the 20%–27% reduction in tensile force reported in previous studies on aging elastomeric ligatures (Ahrari et al., 2010), indicating that the Ca-F O-rings maintain acceptable orthodontic performance requirements. Notably, the 5% group showed a favorable balance between sustained fluoride release and elastic performance *in vitro.*

The coating method is simple and reliable. Using acetone to dissolve the PCL and a short dipping time (1 s) for coating largely preserved the matrix of elastomeric ligature tie. Acetone is a mild solvent that has less effect on polyurethane than other PCL solvents such as dimethyl chloride and chloroform. In addition, the preparation time and protocol are feasible during clinical treatment. Therefore, this study not only provides a platform for future basic studies to optimize therapeutic fluoride dose for WSL prevention but also has clinical translation potential.

Excessive fluoride intake can lead to dental and skeletal fluorosis. According to the NIH Office of Dietary Supplements, the recommended daily fluoride intake for individuals aged 9–18 years and adults is 2–3 mg/day and 3–4 mg/day, respectively, with a tolerable upper intake level (UL) of 10 mg/day (NIH, 1997). In our study, even if a patient were to wear 28 of the 5% Ca-F O-rings simultaneously, the total estimated fluoride exposure would be approximately 1.4 mg/day, well below the UL. Considering local and systemic fluoride levels can vary with saliva flow, oral hygiene, and water consumption, clinicians may further minimize the risk of overexposure by combining regular O-rings with Ca-F O-rings and selectively placing Ca-F O-rings only in areas at higher risk for WSL formation. Additional studies are needed to refine placement strategies and ensure optimal safety and effectiveness in clinical practice.

This study has several limitations. The current coating protocol produced uneven layers on the O-rings, with thicker coatings observed on the inner circumference than on the outer surface. This variation may result from inconsistencies in the original O-ring geometry or rapid solvent evaporation during the drying step, as acetone has a high volatilization rate. For successful clinical translation, more precise coating methods must be developed to achieve uniform PCL coverage with adequate abrasion resistance under oral conditions. Additionally, distilled water was used in this study as the release medium to characterize intrinsic fluoride diffusion kinetics under controlled and reproducible conditions, independent of ion–protein interactions. However, it does not accurately mimic the complex oral environment (e.g., pH cycling, ion and protein content, and saliva viscosity). Future studies should also evaluate the coating durability via scratch (Murase et al., 2020) and toothbrushing abrasion (Dobler et al., 2023), determine the fluoride release in artificial saliva with pH cycling (Abufarwa et al., 2018), and assess treatment efficacy *in vivo*.

In summary, this study showcases the efficacy of employing PCL medium and calcium fluoride as innovative approaches to improve sustained fluoride release, reduce burst effects, and maintain the elastic strength of orthodontic O-rings. These findings hold promising implications for orthodontic treatments, offering a potential solution to prevent WSLs effectively and efficiently.

## Conclusion

The present *in vitro* study explores a potential approach for reducing the risk of WSLs during orthodontic treatment through the development of CaF_2_ coated O-rings. The main findings are as follows:

1. The dip-coating method was effective for applying PCL incorporated with CaF_2_ microcrystal onto O-ring surfaces.
2. The 5% and 10% Ca-F O-ring groups demonstrated sustained fluoride release over an extended period *in vitro.*
3. The Ca-F O-rings maintained their elastic properties after dip-coating modification, indicating material stability within the tested *in vitro* setting.

These findings provide preliminary feasibility-level evidence supporting Ca-F O-rings as a potential WSL-preventive strategy; however, larger-scale studies are necessary to confirm their *ex vivo* performance and to determine their therapeutic efficacy and clinical benefit.

## Data Availability

The raw data supporting the conclusions of this article will be made available by the authors, without undue reservation.
